# Supplementary material for: Contrasting impacts of environmental variability on the breeding biology of two sympatric small procellariiform seabirds in south-eastern Australia
Source: PLoS One. 2021 Sep 13;16(9):e0250916. doi: 10.1371/journal.pone.0250916 (PMC8437294; doi:10.1371/journal.pone.0250916)
Supplement: S1 File — This file contains all the supporting tables. (PDF) [file pone.0250916.s001.pdf]

S1 Table. Results of the GLMM explaining the effect of inter-annual variation of sea surface temperature (SST) on breeding parameters (breeding success (binomial) and phenology (gaussian)) for fairy prions. SST_Summer = average SST in Bass Strait during the summer period (key period for coastal krill; Dec-Feb) preceding the next breeding cycle; SST_PostBreeding = average SST in the post-breeding dispersion area of FP (Feb-April) preceding the next breeding cycle; SST_EarlyBreeding = average SST in Bass Strait during the early stage of the breeding period of FP (Sept-Dec). Nest ID was added as a random effect (1 | Nest ID). Explanatory variables, Akaike Information Criteria (AIC) and Delta AIC (difference in AIC with the best model) are given for each model. Models are ranked according to their respective AIC level.

Response variable	Model	AIC	ΔAIC	LogLik	Weight
Breeding success*	~ SST_Summer + (1 | Nest ID)	198.6	0.00	-96.31	0.38
	~ SST_PostBreeding + (1 | Nest ID)	200.5	1.90	-97.26	0.15
	~ SST_Summer + SST_PostBreeding + SST_EarlyBreeding + (1 | Nest ID)	202.3	3.72	-96.17	0.06
	~ 1 + (1 | Nest ID)	207.3	8.71	-101.66	0.00
	~ SST_EarlyBreeding + (1 | Nest ID)	207.3	8.73	-100.67	0.00
Phenology	~ SST_PostBreeding + SST_Summer + SST_EarlyBreeding + (1 | Nest ID)	700.6	0.00	-344.31	0.664
	~ SST_PostBreeding + (1 | Nest ID)	707.6	6.97	-349.80	0.020
	~ SST_Summer + (1 | Nest ID)	712.0	11.42	-352.02	0.002
	~ SST_EarlyBreeding + (1 | Nest ID)	756.0	55.34	-373.98	0.000
	~ 1 + (1 | Nest ID)	756.4	55.73	-375.18	0.000

*Breeding success corresponds to the breeding success 12 d after hatching (see Results)

S2 Table. ANOVA results of top GLMM for fairy prions (see S1 Table).

	Variable	Sum Squared	F-value
Breeding Success	SST_Summer	7.85	7.85
	SST_PostBreeding	0.06	0.06
	SST_EarlyBreeding	0.22	0.22
Phenology	SST_Summer	627.23	73.65
	SST_PostBreeding	63.69	7.48
	SST_EarlyBreeding	27.71	3.25

S3 Table. Model-averaged coefficients of breeding success and phenology for fairy prions.

	Variable	Estimate	Std. error	z-value	p-value	CI
Breeding Success	Intercept	0.887	0.186	4.728	< 0.001	[0.519 – 1.255]
	SST_Summer	-0.445	0.389	1.138	0.255	[-1.255 – 0.027]
	SST_PostBreeding	-0.205	0.384	0.533	0.594	[-1.346 – 0.469]
	SST_EarlyBreeding	0.039	0.128	0.305	0.761	[-0.276 – 0.515]
Phenology	Intercept	167.563	23.094	7.194	< 0.001	[121.912 – 213.215]
	**SST_Summer**	**2.597**	**0.976**	**2.643**	**0.021**	**[0.671 – 4.523]**
	SST_EarlyBreeding	2.013	1.279	1.814	0.164	[-0.188 – 4.867]
	SST_PostBreeding	2.342	2.183	1.299	0.303	[-1.452 – 7.163]

S4 Table. Results of the GLMM explaining the effect of inter-annual variation of sea surface temperature (SST) on breeding parameters (breeding success (binomial) and phenology (gaussian)) for common diving petrels. SST_Summer = average SST in Bass Strait during the summer period (key period for coastal krill; Dec-Feb) preceding the next breeding cycle; SST_PostBreeding = average SST in the post-breeding migration area of CDP (Dec-Feb) preceding the next breeding cycle; SST_EarlyBreeding = average SST in Bass Strait during the early stage of the breeding period of CDP (July-Sept). Nest ID was added as a random effect (1 | Nest ID). Explanatory variables, Akaike Information Criteria (AIC) and Delta AIC (difference in AIC with the best model) are given for each model. Models are ranked according to their respective AIC level.

Response variable	Model	AIC	ΔAIC	LogLik	Weight
Breeding success	~ SST_Summer + (1 | Nest ID)	61.1	0.00	-26.53	0.87
	~ SST_PostBreeding + SST_Summer + SST_EarlyBreeding + (1 | Nest ID)	69.9	8.83	-28.95	0.01
	~ SST_PostBreeding + (1 | Nest ID)	220.3	159.18	-106.13	0.00
	~ SST_EarlyBreeding + (1 | Nest ID)	251.6	190.51	-121.79	0.00
	~ 1 + (1 | Nest ID)	257.1	196.06	-125.57	0.00
Phenology	~ SST_PostBreeding + SST_Summer + SST_EarlyBreeding + (1 | Nest ID)	887.5	0.00	-437.77	1
	~ SST_Summer + (1 | Nest ID)	975.5	88.02	-483.77	0
	~ SST_PostBreeding + (1 | Nest ID)	1026.6	139.12	-509.32	0
	~ SST_EarlyBreeding + (1 | Nest ID)	1078.5	190.73	-535.13	0
	~ 1 + (1 | Nest ID)	1083.2	195.65	-538.59	0

S5 Table. ANOVA results of top GLMM for common diving petrel (see S4 Table).

	Variable	Sum Squared	F-value
Breeding Success	SST_Summer	25.39	376.56
	SST_PostBreeding	0.16	2.33
	SST_EarlyBreeding	0.04	0.63
Phenology	SST_Summer	31112.9	350.21
	SST_PostBreeding	2614.5	29.43
	SST_EarlyBreeding	8139.7	91.62

S6 Table. Model-averaged coefficients of breeding success and phenology for common diving petrel.

	Variable	Estimate	Std. Error	z-value	p-value	CI
Breeding Success	Intercept	0.292	0.019	14.606	< 0.001	[0.253 – 0.331]
	**SST_Summer**	**-0.359**	**0.056**	**6.392**	**<0.001**	**[-0.420 – -0.311]**
	SST_PostBreeding	0.011	0.064	0.182	0.856	[-0.207 – 0.486]
	SST_EarlyBreeding	-0.007	0.056	0.126	0.899	[-0.462 – 0.266]
Phenology	Intercept	243.34	0.884	262.395	< 0.001	[232.6 – 236.1]
	**SST_Summer**	**71.04**	**6.138**	**11.453**	**< 0.001**	**[58.9 –83.2]**
	**SST_EarlyBreeding**	**-75.86**	**7.925**	**9.471**	**< 0.001**	**[-91.6 – -60.2]**
	**SST_PostBreeding**	**83.83**	**9.410**	**8.815**	**< 0.001**	**[65.2 – 102.5]**
